# Early vs. late coronary angiography in cardiac arrest improves survival and preserves renal function: some confounding factors to consider

**DOI:** 10.1186/s13054-019-2676-2

**Published:** 2019-11-27

**Authors:** Sébastien Redant, Yael Langman, Xavier Beretta-Piccoli, David De Bels, Rachid Attou, Patrick M. Honore

**Affiliations:** 1 ICU Department, Centre Hospitalier Universitaire Brugmann, Brugmann University Hospital, Place Van Gehuchtenplein 4, 1020 Brussels, Belgium; 2 Pediatric Intensive Care Department, Hôpital Universitaire des Enfants Reine Fabiola, Université Libre de Bruxelles, Brussels, Belgium

Rundgren et al. studied the evolution of renal function in postcardiac arrest as a function of hypothermia and timing of angiography (early vs. late). They have concluded that early coronary angiography (CA) does not increase the risk of developing acute kidney injury (AKI). They also showed a significant difference in survival at 6 months [1]. The authors also noted that lactate at admission was statistically significantly higher in the AKI group [1]. This leads us to question whether these patients had more severe shock or hemodynamic instability and in turn consider whether there was a potential selection bias, with these patients receiving delayed CA due to the need to be stabilized before proceeding. A recent study has shown that an increased number of incompletely revascularized lesions was associated with a greater risk of intra-hospital mortality and poorer neurological outcome in patients after cardiac arrest [2]. The result of the CA and the success of the revascularization procedure should be potential confounders to consider in the AKI risk assessment. It is indeed very elegant to postulate that early CA is accompanied by a recovery of cardiac function that results in improved renal function despite concurrent contrast administration. Renal function strongly depends on renal interstitial pressure and arteriovenous pressure difference. The combination of significant vasodilation from postarrest ischemia-reperfusion with venous congestion, low cardiac output, and activation of a systemic inflammatory response leads to multiple organ dysfunction where AKI is prominent. Increased central venous pressure has been shown to be the most important hemodynamic factor driving worsening renal function in decompensated patients with advanced heart failure [3]. In septic patients, venous congestion has been associated with an increased likelihood of new or persistent acute kidney injury, particularly when combined with a low diastolic blood pressure, a marker of generalized vasodilatation [4]. We would like to see the objective data regarding cardiac function including pre- and postcoronary angiography. It has also been shown, but in the context of sepsis, that recovery of renal function is inconsistent after normalization of hemodynamics or could take a much longer time to recover [5]. In conclusion, we find this study very interesting in assessing the impact of early vs. late angiography on renal function. Nevertheless, this study lacks to show the input of potential confounders of renal injury such as the severity of the coronary lesions, the level of success of the CA, and more precise hemodynamics data.

## Data Availability

Not applicable.

## Abbreviations

CA: Coronary angiography
AKI: Acute kidney injury
